# Traditional bullying and cyberbullying among children and adolescents in Germany – Cross-sectional results of the 2017/18 HBSC study and trends

**DOI:** 10.25646/6902

**Published:** 2020-09-16

**Authors:** Saskia M. Fischer, Nancy John, Wolfgang Melzer, Anne Kaman, Kristina Winter, Ludwig Bilz

**Affiliations:** 1 Brandenburg University of Technology Cottbus-Senftenberg Faculty of Social Work, Health Care and Music, Institute of Health; 2 Technische Universität Dresden Institute of Education; 3 University Medical Center Hamburg-Eppendorf Center for Psychosocial Medicine, Department of Child and Adolescent Psychiatry, Psychotherapy and Psychosomatics; 4 Martin Luther University Halle-Wittenberg Medical Faculty, Institute of Medical Sociology; 5 Brandenburg University of Technology Cottbus-Senftenberg Faculty of Health Sciences

**Keywords:** BULLYING, CYBERBULLYING, FREQUENCY, PREVALENCE, TRENDS, SCHOOL, VIOLENCE

## Abstract

Bullying is a specific form of violence that can potentially lead to numerous and long-term negative health implications. Despite consistent coverage in the media, particularly on cyberbullying, as of yet there are only few representative findings on the frequency of (cyber)bullying in Germany. This article analyses how widespread bullying and cyberbullying were at schools in Germany in 2018, what differences exist between girls and boys, age groups and various types of schools, and changes in bullying trends between 2002 and 2018. Our findings are based on an analysis of the data provided by the 2017/18 cycle (N=4,347 students, 53.0% female) and previous cycles of the German Health Behaviour in School-aged Children (HBSC) study. In the 2018 cycle, boys reported having bullied other children more frequently than girls, but were bullied just as often. 15-year-olds reported having bullied other children more frequently than 11- to- 13-year-olds but reported being bullied less frequently. Students at grammar schools (Gymnasium) least frequently reported any involvement in bullying. Only few children and adolescents reported cases of cyberbullying. Compared to all previous survey years, 2018 saw the lowest number of children that reported having bullied others. However, reports of having been bullied have remained almost stable. The findings highlight the need for evidence-based prevention and intervention anti-bullying programmes and measures across all types of general education schools and age groups.

## 1. Introduction

Bullying describes a specific and repeated form of violence exerted precisely to hurt others. The imbalance of power between bullies and their victims makes it hard for the latter to defend themselves from being bullied [1]. Bullying is an attack on the fundamental right of children and adolescents to respect, safety and physical integrity, as well as to grow up in an environment free of violence. Bullying can entail multiple and severe consequences. It can increase the risk of students underperforming academically or attempting to avoid school altogether, but also makes them more susceptible to depression, suicidality and psychosomatic disorders [2–5]. Longitudinal studies indicate that being bullied during childhood can negatively impact mental health even into adolescence and adulthood [6–8]. Overall, experiences of bullying are one of the chief risk factors for mental disorders [9]. The bullying of children and adolescents who are victimised because they belong to a cultural, religious or sexual minority (bias-based bullying) can have particularly negative impacts [10, 11]. There are three groups: students who bully others, those who suffer bullying, and those who both bully and are bullied. The latter run a particularly high risk of suffering health impacts [12]. Furthermore, uninvolved students that neither actively bully nor suffer bullying, also play an important role, for example, when they stand by the victims or, otherwise, support the bully [13].

**Info box:**

**German secondary school system**

This paper includes terminology specific to the German secondary school system, whereby students can attend different schools that vary in their level of academic and/or vocational focus. In general, a Hauptschule is attended by students aged 10 to 16 and offers a basic general education, a Realschule provides a more extensive education for students aged between 10 and 16. A Gymnasium teaches students aged between 10 and 19, provides an in-depth general education and is focused on preparing students for higher education.

Gemeinschaftschulen are secondary education schools, primarily for students aged 10 to 16, where students learn together and are able to sit the same qualifications offered in the three other school types (Hauptschule, Realschule and Gymnasium).

Bullying can take different forms. Generally, studies differentiate between three types: name-calling and insulting (verbal bullying), hitting and kicking (physical bullying), and socially excluding the victim and spreading rumours (relational bullying). Verbal and relational bullying in particular can also be practised online (cyberbullying). We call non-online forms of bullying ‘traditional bullying’ [14].

Suffering online forms of bullying is one of the greatest risks adolescents run when they use the internet [15, 16]. Cyberbullying’s specific modalities (in particular the greater levels of anonymity, widespread use and easy access) contribute to victims feeling less at ease and out of place at school, and increase their risk of developing mental disorders [17, 18]. International studies have thereby shown that relatively few children and adolescents suffer cyberbullying, in particular compared to traditional forms of bullying [14, 15, 17, 19, 20]. According to these studies, far fewer people suffer cyberbullying than media reports would suggest [21, 22]. Representative findings on the prevalence of cyberbullying among students in Germany are rare [20]. For Germany, the representative findings of the 2018 Youth, Information and Media study (JIM) suggests that around one in five adolescents have encountered cyberbullying, which would indicate that cyberbullying is widespread in Germany. However, the study did not collect data on non-online bullying [16].

Meaningful group differences regarding the prevalence of (cyber)bullying exist. Boys appear to bully more than girls [23] – both in terms of cyberbullying [16, 24] and traditional forms of bullying [5, 25, 26, 27] – although the literature provides no consistent results on the differences between the sexes with regard to cyberbullying [28]. Studies mostly show that girls encounter bullying – both traditional bullying [5, 26] and cyberbullying [28] – more often than boys [24]. There are, however, also other findings reporting that boys make up a higher percentage of those students that suffer bullying [23]. Moreover, studies show differences depending on the type of bullying. Boys use verbal and physical bullying more often than girls [29] and also suffer these forms of bullying more frequently [25]. The differences for physical bullying are thereby greater than for verbal bullying [27, 29]. On the other hand, girls appear to both use and suffer relational bullying more frequently than boys, whereby the differences between the sexes here are far smaller than for verbal and physical bullying, with some studies failing to detect such a difference at all [25, 27, 29].

Many studies also indicate school type (see Info box) as a relevant factor for bullying and being bullied. Findings from the German-speaking region show a far greater involvement of lower secondary school (Hauptschule) students in bullying than students at grammar schools (Gymnasien) [30] and intermediate secondary schools (Realschulen) [16, 26]. For a further potential factor, age, studies have shown that bullying occurs particularly often at the middle school-age range (classes six to nine) [5, 27], whereby German-language studies so far have not reported systematic empirical differences for this age interval [26, 27, 30].

Recent years have seen a considerable drop in bullying both internationally and in Germany [26, 30, 31]. In studies from Germany, the number of girls and boys that report having bullied other children is decreasing [26, 30]. Figures for being bullied are possibly only declining for boys [30]. Furthermore, the decrease in the number of students who bully others [30] or who are bullied is particularly evident among elder adolescents [26].

Our analyses will seek to find out whether the declining trends for bullying and experiences of being bullied have continued in 2018 and whether differences can be discerned regarding sex and age groups. We also conduct a closer analysis of the frequency of bullying and cyberbullying in 2018 regarding overall bullying and experiences of being bullied, the different bullying types, as well as potential differences with regard to sex, age and type of school. Our analyses build on the representative data provided by the 2017/18 cycle of the Health Behaviour in School-aged Children (HBSC) study.

## 2. Methodology

### 2.1 Sample design and study implementation

To answer the outlined research questions, we used the 2018 HBSC data for Germany. The data comprised responses given by students from general education schools in Germany from years five, seven and nine. Participating schools were selected by stratified random sampling (stratified by federal state and type of school). Interviews were conducted during lessons via written questionnaires. Participation by schools and students was voluntary and subject to students and their parents/legal guardians providing written consent. The ministries of education and cultural affairs of all federal states approved the study. A total of 4,347 students (53.0% female) at 146 general education schools of all types in Germany participated in the study. The interviewed students were 11 (32.2%), 13 (32.6%) and 15 years old (35.2%) when surveyed. The article by Moor et al. in this issue of the Journal of Health Monitoring contains detailed information on the HBSC study and its methodology.

### 2.2 Surveying instruments

#### Bullying in general

Bullying at school was surveyed using the Revised Olweus Bully/Victim Questionnaire (OBVQ) [32]. The questionnaire begins with a short age-appropriate definition of bullying, highlighting the key elements: bullying occurs repeatedly, aims to hurt others and is based on a power imbalance. Students were then asked whether they had bullied others in recent months (How often during recent months have you taken part in bullying at school?) or had been bullied (How often in recent months have you been bullied at school?). Frequencies were recorded based on a five-tier scale (no bullying during the last couple of months, once or twice, two to three times per month, about once per week, several times per week). These two questions served to collect data on general experiences of bullying during the reference period, whereby bullying was considered to have occurred if students answered at least ‘two to three times per month’.

#### Types of bullying

The OBVQ [32] also asked students how often they had participated in, or fallen victim to, seven specific forms of bullying during recent months. We can divide these seven types of bullying into three separate categories: physical, verbal and relational bullying. Each type of bullying is thereby represented by a different number of items: physical bullying by one (hitting), verbal bullying by four (name-calling and insulting other students due to their ethnicity, religion or sexual orientation) and relational bullying by two (socially excluding others, lying and spreading rumours). The answer options provided were the same as the five described above. Experience with a particular type of bullying was considered to be present if students had either carried out or experienced at least one of the forms of bullying at least two to three times per month.

#### Cyberbullying

To survey cyberbullying, we used an adapted version of the revised OBVQ [32]. With one item each, students were asked about cyberbullying (How often have you bullied someone online during the last couple of months?) and whether they had been cyberbullied (How often have you been bullied online during the last couple of months?). Examples of cyberbullying, such as writing mean messages, emails, text messages or posts, creating websites to make fun of someone, or sending unflattering pictures, were provided. Data were collected and categorised using the methods described above both for general experiences of bullying and being bullied as well as experiences differentiated according to the type of bullying.

#### Control variables

To analyse group differences, we collected data on sex, age group and type of school. Students self-reported sex and age in the questionnaires. Age was categorised by the HBSC Data Management Centre (University of Bergen) during data cleansing, allowing the differentiation of three age groups (11-, 13- and 15-year-olds). To better reflect the situation in all federal states, the survey staff defined four categories for school types: lower, intermediate and grammar schools and mixed school types (e.g. comprehensive schools (Gemeinschaftsschulen), see Info box).

### 2.3 Statistical analysis

To analyse the frequency of bullying and cyberbullying and the differences between groups for the survey year 2018, a typology was developed that differentiates between students that bully, those that are bullied, and those who fall into both categories (students who both bully and are bullied), as well as uninvolved students that neither bully nor are bullied. This typology was used on bullying in general, types of bullying, as well as cyberbullying. Cross tables and chi-square tests with post-hoc analysis were used to analyse potential differences between groups.

Based on the described typology of bullying roles, the trends for bullying frequency between 2002 and 2018 were analysed by logistic regression with robust standard errors that correct for non-normal distribution and a lack of independence of data (heteroscedasticity). For analysis, one of the four categories of the typology was opposed with the other three (dummy coding) and then analysed as an independent variable in individual regression analyses. The survey year along with interaction effects between survey year and sex as well as survey year and age group (11-, 13- and 15-year-olds) were incorporated into the analysis as predictors.

All calculations were carried out using a weighting factor that corrects deviations within the sample from the basic population structure (students in Germany) with regard to type of school, age and sex. Cleansing of the raw data set from the German HBSC study was conducted centrally by the HBSC Data Management Centre (University of Bergen). The analyses presented here used SPSS 22 as well as Mplus 8.3. The level of significance of the analysis at the data collection point (2018) was set at p < 0.05. Trend analyses use a high number of individual comparisons. For these calculations, the level of significance was therefore set at a more conservative p < 0.001.

## 3. Results

### 3.1 Bullying: frequency and differences between groups

Table 1 shows the figures for the different bullying roles according to typology (section 2.3). The vast majority of students reported that they had neither bullied fellow students nor been bullied themselves (uninvolved: 86.7%). Being bullied is an experience reported far more frequently (8.3%) than active bullying (3.9%). Only few students find themselves in the double role of both bullying others and being bullied (1.1%).

Chi-square test results show differences in the level of bullying between girls and boys, between 11-, 13- and 15-year-olds, and also with regard to type of school (Table 1). Boys self-report participating in bullying more often than girls, and also more often find themselves in the double role of bully and victim. In terms of falling victim to bullying, no differences between the sexes were found. 15-year-olds are considerably more often bullies than 11- and 13-year-olds, but are less often bullied than 13-year-olds. Students at grammar schools are far less often involved in any bullying role than students at other types of school. For students at lower secondary schools, intermediate secondary school and schools that offer various types of school leaving certificates (mixed school types), no differences regarding bullying experiences were found.

### 3.2 Physical, verbal and relational bullying: frequency and differences between groups

Analyses for each of the individual types of bullying (physical, verbal and relational bullying) again applied the developed typology of bullying roles (Table 2). Boys reported physically and verbally bullying others more often than girls, with no differences by sex found for relational bullying. Boys also reported being physically bullied more often than girls, while girls suffered verbal and relational bullying considerably more often than boys. 15-year-olds use verbal bullying considerably more often than 11- and 13-year-olds, while there are particularly low figures for relational bullying among 11-year-olds. When differentiated by type of bullying and by age group, the findings differ from those found for bullying in general: overall, 15-year-olds less frequently report being bullied (Table 1); yet they do not suffer considerably less relational and verbal bullying than 11- and 13-year-olds. Findings for the overall prevalence of bullying, according to which students at grammar schools are least involved in bullying events (Table 1), are confirmed for most types of bullying and bullying roles. Students at grammar schools nonetheless report actively participating in relational bullying just as often as students at other types of schools. Moreover, the small group of students that both exert and suffer physical, verbal and relational bullying is found just as often in grammar schools as at other types of school. While bullying occurs less frequently at grammar schools compared to other types of school, it does still occur in certain forms. No differences were found between lower secondary schools, intermediate secondary schools, as well as schools offering different types of leaving certificates regarding the reported roles of bully and victim, and types of bullying.

### 3.3 Cyberbullying: frequency and differences between groups

We also applied the typology of bullying roles to analyse the prevalence of cyberbullying. The results are shown in Table 3. 1.3% of students reported having actively bullied fellow students online. 2.0% reported having been bullied online and 0.6% stated they experienced cyberbullying in both roles. The vast majority of students reported not having had any experiences with cyberbullying (96.0%).

The analysis of differences between groups (Table 3) showed that girls reported being bullied online more frequently than boys. Unlike for bullying in general (Table 1), no differences by sex were found for actively bullying others online. The proportion of students that were bullied online, as well as of those who both bully and are bullied, is similarly high for the three age groups of 11-, 13- and 15-year-olds. However, elder adolescents participate in cyberbullying considerably more often than their younger counterparts. Students at grammar schools self-report significantly lower figures for bullying others online and are also bullied significantly less frequently than students at schools that offer various types of school leaving certificates. They are also less likely to be in the double role of both perpetrator and victim of online bullying than students at intermediate secondary schools. No differences between lower secondary schools, intermediate secondary schools and schools with multiple types of leaving certificates were found regarding the proportion of students in the different cyberbullying roles.

### 3.4 Prevalence of bullying between 2002 and 2018

The analyses of the trends for bullying figures between 2002 and 2018 are based on the typology of bullying in general (section 3.1). This does not allow for an analysis of cyberbullying, for which data was only first collected in 2018.

Table 4 shows the distribution of bullying roles between 2002 and 2018. Logistic regression analysis with robust standard errors show that in 2018 fewer students reported having bullied others compared to all previous survey years. When examining the proportion of students that reported being a victim of bullying, the figure for 2018 is only lower in comparison to 2006 and has otherwise remained stable. The apparent, slight percentage increase for this group between 2014 and 2018 is therefore not statistically significant. In 2018, the group that reports having bullied others and having been bullied is considerably smaller than in 2002 and 2006, but has remained stable since. Overall, a smaller number of students reported having actively taken part in bullying in 2018 than in 2002, 2006 and 2010. However, between 2014 and 2018 bullying figures did not decrease significantly.

Due to the differences that the 2018 data revealed between the groups of girls and boys as well as between the three age groups, a further analysis focused on whether bullying had also developed differently depending on sex and age group. Further regression analyses therefore set an interaction effect between sex (or age group) and the corresponding surveying year and used this as a predictor next to the respective main effect (sex or age group). 2018 again served as a benchmark for comparisons. The analyses for age categories used the group of 15-year-olds as a benchmark. None of the interaction terms based on the sex of respondents was statistically significant for any of the bullying groups. The development of the trend therefore did not differ for girls or for boys. The trend for the distribution of bullies, bullied and students who find themselves in both roles does not differ between age groups. Significant interaction effects do exist for some groups such as the uninvolved (between the group of 11-year-olds and the group of 15-year-olds, and the years 2006, 2010 and 2018). However, as these are merely isolated findings that are unrelated to the most recent developments between 2014 and 2018, we have not interpreted these findings.

Diverging developments in the prevalence of bullying by type of school were not calculated because changes to education policy have led to considerable shifts in the German education system in recent years. The number of lower secondary schools, for example, has more than halved, dropping from 7,657 in the 2001/02 school year [33] to 3,399 in the 2017/18 school year [34]. These developments too severely limit the reliability of findings for trends specific to school type.

## 4. Discussion

### 4.1 Prevalence of bullying and cyberbullying

In 2018, around 13% of participating students in Germany reported having had direct experiences of bullying, either because they had bullied other students, suffered bullying themselves or been active in both bullying roles. Analyses by type of bullying reveal that a particularly large number of children and adolescents were involved in incidents of verbal and relational bullying. Only relatively few children and adolescents reported having physically bullied someone or being physically bullied. The reported figures do not make it possible to differentiate between traditional and cyberbullying. To gain an overview of levels of cyberbullying among 11-, 13-, and 15-year-old students in Germany, the 2018 survey was the first to ask explicitly about experiences of cyberbullying. Around 4% reported either having bullied fellow students or being bullied online. While this makes cyberbullying a relevant problem among students in Germany, the phenomenon is nowhere near as widespread as its prominence in the media and public discourse would suggest [21, 22]. German figures for the prevalence of cyberbullying are similar to those found in international results [14, 19, 20]. However, our results contradict those of the JIM study, which was conducted in Germany [16] and reported far higher levels of cyberbullying. When interpreting these different results, it will be necessary to consider the underlying definitions of bullying and the ways in which it is surveyed. Whereas the JIM study records even single incidents as cyberbullying, for example, our study applied the definition used by Olweus [1], according to which bullying is a repeated experience.

Cyberbullying is often compared to the prevalence of traditional forms of bullying, whereby all forms of bullying that are not explicitly cyberbullying are then considered as traditional forms of bullying. Children and adolescents, however, frequently do not distinguish between online and offline environments in the same way as adults do [35]. Verbal and relational bullying in particular can also be used online. When we ask adolescents about their experiences with different bullying strategies, it is therefore not always clear whether such bullying occurs online or offline. Correspondingly, this article does not compare overall bullying and cyberbullying frequencies. Instead, we consider cyberbullying as a subset within overall bullying frequencies and the reported frequencies for individual types of bullying.

In a similar fashion to the most recent HBSC survey cycles, we based our distinction of the groups analysed (those who bully, are bullied and those active in both roles) on content-based theoretical considerations. Next to this common form of categorisation, there are also empirical approaches to defining groups. A comparison of both approaches shows that the theoretical approach chosen here can lead surveys to overestimate bullying frequency [36]. What is more, cleanly distinguishing between roles is far more difficult, particularly with regard to cyberbullying. In the case of cyberbullying it is rare for an individual to exclusively be a bully or a victim without ever having experienced the role of the other [36]. Moreover, bullying roles appear to be far more complex in cyberbullying than in traditional bullying [37]. The roles described here should therefore be seen as prototypical descriptions that are potentially more complex in practice.

### 4.2 Differences between groups in the prevalence of (cyber)bullying

Boys report bullying others more often than girls. German HBSC data from 2014 had already highlighted this difference between sexes [26]. More recent analyses, however, now show that differences also exist by type of bullying. Girls are just as likely to be actively involved in relational bullying and cyberbullying as boys, a finding corroborated by existing research [25, 27, 29]. However, there are no plausible justifications to explain the differences between the sexes [28]. Entrenched gender role models could lead boys to rely more on physical forms of bullying, while the manner in which girls bully is based more around social relationships [23, 25, 29].

Girls reported suffering cyber and relational bullying more often than boys. Boys, in turn, more frequently reported being physically bullied. However, if we look at bullying in general without differentiating between types of bullying, girls are bullied just as often as boys. This differs from the 2014 HBSC data [26]: findings indicated that more girls had been bullied than boys during this year. It appears bullying has now reached similar levels for both sexes.

Findings on the levels of bullying at different types of schools and years basically confirm the findings of existing German language research. As was the case in previous HBSC study cycles for Germany, in this survey students at grammar schools once again reported that they rarely bullied others or were bullied themselves [26, 30]. Contrary to the survey cycle 2013/14, meaningful differences between intermediate secondary schools and lower secondary schools, as well as between lower secondary schools and schools that offer various types of leaving certificates were no longer detected in 2017/18. A descriptive comparison of data leads to the conclusion that this is probably mainly due to the fact that students at lower secondary schools in 2014 bullied other students far more frequently (10.7% [26]) than in 2018 (5.5%). We can only speculate on the reasons for this decrease. Any attempt at an interpretation needs to consider structural change that has occurred. For example, developments in education policy have led to a considerable decrease in the number of lower secondary schools in the period up to 2018. This type of school is therefore represented by a much smaller number of students (around 8.5%) in the survey year 2018 compared to the survey year 2014 (around 15% [26]), which makes random fluctuations in the data more likely.

Our analysis differentiates between types of bullying and thereby expands German language research which has only considered differences by types of school. Students at grammar schools, as we have seen, are overall less involved in bullying and are also bullied less than students at other school types, yet levels of relational bullying are comparable. For those in the double role of bully and victim, no differences between types of school exist and this applies for all types of bullying. This small group, which is, however, particularly affected by the negative consequences of bullying [12], therefore exists at all types of school, a fact which highlights the importance of evidence-based anti-bullying measures, including at grammar schools, and the need to train teachers and students on how to prevent and halt the prevalence of bullying.

Unlike HBSC data from the survey cycle 2014, as well as earlier surveys with students in Germany [26, 27, 30], considerable differences between age groups existed in 2018. Findings indicate that 15-year-olds are more involved in bullying, but are bullied less than 11- and 13-year-olds. These findings could indicate that students bully others or are bullied outside of their year and age group (for example during breaks). This assumption highlights the need for co-operative school-wide approaches to prevention and intervention that should involve all students at a particular school.

To interpret the data on individual types of bullying, it is important to take into account the specificities of the surveying instrument. The questionnaire used to collect data on bullying is an established instrument that is frequently used internationally and has good psychometric characteristics [38]. However, it differentiates bullying experiences based on theoretical considerations and not empirically grounded. Data for the individual types of bullying are collected using different numbers of items and, potentially, this could influence the results. In addition, the instrument applied in this survey cannot provide findings on currently discussed bullying concepts such as bias-based bullying [10, 11].

### 4.3 Development of levels of bullying between 2002 and 2018

An analysis of trends shows that in 2018 fewer students reported having bullied other students than in all survey years from 2002 to 2014. With regard to the other bullying roles, we can assume a stabilisation of the frequency of bullying. Across all bullying roles and age groups, the trend for girls and boys is thereby similar. This finding contradicts the results of the HBSC survey cycle from 2014 [26], as well as a further survey on bullying conducted with students in Saxony [30]. Importantly, however, the findings of these studies rely on different forms of statistical analysis. A statistical control through robust standard errors as well as a conservative choice of level of significance were not applied by these past studies.

In general, the finding of a decreasing frequency of bullying is in line with other national and international findings [26, 30, 31]. In recent years, however, a decrease was recorded not only for active bullying but also for victims of bullying [26]. In 2018, a decreasing number of students reported having bullied other students, while figures for being bullied did not continue to drop. This raises the question as to whether reporting effects possibly explain decreasing bullying figures. Intense coverage on the issue of bullying in the media could raise students’ awareness of the fact that actively bullying others is not socially acceptable and lead to students being reluctant to report having actively bullied others, even in anonymous surveys. What people believe is socially acceptable, however, would have a smaller effect on reporting having been bullied, and this could explain the disparity in the trends for active bullying and being bullied.

Alternatively, the stable decrease in the number of students who bully other students could reflect an actual decrease and be a consequence of greater efforts to tackle bullying at school. The few students that continue to bully others in spite of more comprehensive prevention and intervention strategies could be students with more fixed behaviour patterns and who therefore are particularly hard to reach through interventions. It is possible that these students bully a number of other students. A small proportion of bullies would then produce a relatively larger proportion of students that are bullied and this would potentially explain the diverging trends.

As it remains impossible to say which of these explanations best fits the results found, it could also be that a combination of the two theories conclusively explains the findings.

### 4.4 Conclusions

Overall, the decrease or at least stabilisation at a low level of bullying frequencies highlight the importance of proactive anti-bullying interventions. In spite of regressive trends, over one in seven students (both female and male) continues to be involved in bullying. Presumably, therefore, in every single class in Germany, there are children who suffer bullying. The development, evaluation and implementation of school-wide and long-term effective anti-bullying strategies and programmes should therefore be expanded to protect students from bullying and its severe health implications [2–9, 12]. Teachers are key in this regard. They should be supported in their capacity to recognise bullying and react effectively [39, 40]. In particular with regard to the relatively stable number of students that are being bullied, it is important to ensure teachers are made aware of the effects of bullying and encouraged to reach out to external co-operation partners, such as anti-bullying support centres [41].

### Key statements

- In 2018, 13.3% of students stated that they had been involved in bullying incidents. 3.9% of students reported either having been bullied online or bullying other children and adolescents online.
- With the exception of relational bullying, where no differences by sex were found, for all other types of bullying, boys reported having bullied fellow students more frequently than girls.
- Girls reported having been bullied online more frequently than boys and suffered more from relational and verbal bullying. Boys, in turn, more frequently reported involvement in physical bullying than girls.
- Compared to previous years, fewer students reported bullying other children and adolescents in 2018. Compared to 2014, the proportion of students that reported being bullied has remained stable.
- The downward trend in the number of children and adolescents who reported being bullied in the 2002 to 2018 reporting period is similar for girls and boys.

## Figures and Tables

**Table 1:** Bullying typology by sex, age and type of school (n=2,118 girls, n=2,079 boys) Source: 2017/18 German HBSC study

Uninvolved (%)		Bully (%)	Suffered bullying (%)	Double role bully and bullied (%)
**Total (N=4,197)**	**86.7**	**3.9**	**8.3**	**1.1**
**Sex (n=4,196)**				
Girls	88.9_a_	1.8_b_	8.6	0.8_c_
Boys	84.5_a_	6.0_b_	8.0	1.5_c_
**Age group (n=4,158)**				
11 years	87.8	2.3_d_	9.0	0.8
13 years	85.4	3.6_e_	9.3_f_	1.7
15 years	86.9	5.5_d,e_	6.8_f_	0.8
**Type of school (n=4,197)**				
Lower secondary schools	81.2_g_	5.5_j_	11.0_m_	2.3_p_
Intermediate secondary schools	83.6_h_	4.7_k_	9.6_n_	2.1_q_
Grammar schools	91.0_g,h,i_	2.4_j,k,l_	6.0_m,n,o_	0.7_p,q_
Mixed school type	84.9_i_	4.6_l_	9.6_o_	0.9

Lower case letters indicate significant differences between subgroups in post-hoc analysis (p < 0.05), whereby the differences are significant between subgroups with the same letters. Post-hoc analyses were adjusted for multiple tests (Bonferroni adjustment). Values in lines just over or under 100% are due to the rounding of values after the decimal point.

**Table 2:** Physical, verbal and relational bullying by sex, age and type of school (n=2,077 girls, n=2,044 boys) Source: 2017/18 German HBSC study

Physical bullying (%)					Verbal bullying (%)				Relational bullying (%)			
Uninvolved		Bullies	Suffered bullying	Double role*	Uninvolved	Bullies	Suffered bullying	Double role*	Uninvolved	Bullies	Suffered bullying	Double role*
**Total**	**95.0**	**1.5**	**3.0**	**0.5**	**82.8**	**5.3**	**8.2**	**3.7**	**86.3**	**3.3**	**8.4**	**2.0**
**Sex**				(n=4,121)				(n=4,092)				(n=4,107)
Girls	96.9_a_	0.6_b_	2.2_c_	0.3_d_	84.7_e_	3.0_f_	9.5_g_	2.8_h_	84.6_i_	3.0	10.2_j_	2.2
Boys	93.0_a_	2.3_b_	4.0_c_	0.7_d_	80.7_e_	7.7_f_	6.9_g_	4.6_h_	88.0_i_	3.6	6.5_j_	1.8
**Age group**				(n=4,084)				(n=4,057)				(n=4,068)
11 years	95.3	1.2	3.2	0.3	88.2_m,n_	2.4_o,p_	7.7	1.7_r.__s_	89.9_t,u_	1.6_v.__w_	7.2	1.3_x_
13 years	93.6_k_	1.6	4.2_l_	0.6	81.9_m_	5.0_o,q_	9.0	4.1_r_	85.1_t_	3.6_v_	9.4	2.0
15 years	96.0_k_	1.5	1.9_l_	0.6	78.5_n_	8.3_p,q_	8.0	5.3_s_	84.3_u_	4.4_w_	8.6	2.7_x_
**Type of school**				(n=4,120)				(n=4,093)				(n=4,107)
Lower secondary schools	94.0_y_	2.0	3.5	0.5	80.1_ee_	7.1_hh_	9.1	3.8	84.2	4.3	9.8	1.8
Intermediate secondary schools	93.4_z_	2.1_bb_	3.7_cc_	0.8	78.7_ff_	6.9_ii_	11.2_jj_	3.3	83.6_ll_	3.9	10.0_nn_	2.5
Grammar schools	97.0_y.z.aa_	0.8_bb_	1.9_cc,dd_	0.4	86.6_ee,ff,gg_	3.9_hh,ii_	5.9_jj,kk_	3.7	88.9_ll,mm_	2.9	6.6_nn_	1.6
Mixed school type	93.8_aa_	1.8	3.9_dd_	0.5	81.4_gg_	5.6	9.0_kk_	4.0	85.2_mm_	3.2	9.2	2.4

Lower case letters indicate significant differences between subgroups in post-hoc analysis (p< 0.05), whereby the differences between subgroups with the same letters are significant. Post-hoc analyses were adjusted for multiple tests (Bonferroni adjustment). Values in lines just over or under 100% are due to the rounding of values after the decimal point.

* Double role bully and bullied

**Table 3:** Cyberbullying typology by sex, age and type of school (n=2,108 girls, n=2,045 boys) Source: 2017/18 German HBSC

Uninvolved (%)		Bullies (%)	Suffered bullying (%)	Double role bully and bullied (%)
**Total (N=4,153)**	**96.0**	**1.3**	**2.0**	**0.6**
**Sex (n=4,154)**				
Girls	95.9	1.0	2.5_a_	0.5
Boys	96.1	1.6	1.5_a_	0.7
**Age group (n=4,113)**				
11 years	97.4_b_	0.6_c_	1.7	0.2
13 years	95.8	0.9_d_	2.4	0.8
15 years	95.1_b_	2.4_c,d_	1.7	0.8
**Type of school (n=4,153)**				
Lower secondary schools	95.7	1.5	2.0	0.8
Intermediate secondary schools	94.5_e_	1.7	2.5	1.3_i_
Grammar schools	97.8_e,f_	0.7_g_	1.2_h_	0.3_i_
Mixed school types	94.8_f_	1.8_g_	2.8_h_	0.6

Lower case letters indicate significant differences between subgroups in post-hoc analysis (p< 0.05), whereby the differences between subgroups with the same letters are significant. Post-hoc analyses were adjusted for multiple tests (Bonferroni adjustment). Values in lines just over or under 100% are due to the rounding of values after the decimal point.

**Table 4:** Bullying typology over time (n=13,885 girls, n= 3,688 boys) Source: 2001/02–2017/18 German HBSC study

Survey year	Uninvolved (%)	Bullies (%)	Suffered bullying (%)	Double role bully and bullied (%)
2002 (n=5,554)	73.7_a_	13.2_d_	9.5	3.7_i_
2006 (n=7,166)	77.3_b_	8.8_e_	11.2_h_	2.7_j_
2010 (n=4,974)	81.4_c_	8.4_f_	8.6	1.6
2014 (n=5,682)	83.2	7.5_g_	7.8	1.4
2018 (n=4,197)	86.7_a,b,c_	3.9_d,e,f,g_	8.3_h_	1.1_i,j_

Lower case letters indicate significant differences between a specific year and 2018 for the respective bullying role (p < 0.001).
